# Reducing Drinking Among People Experiencing Homelessness: Protocol for the Development and Testing of a Just-in-Time Adaptive Intervention

**DOI:** 10.2196/15610

**Published:** 2020-04-16

**Authors:** Michael S Businelle, Scott T Walters, Eun-Young Mun, Thomas R Kirchner, Emily T Hébert, Xiaoyin Li

**Affiliations:** 1 Oklahoma Tobacco Research Center Stephenson Cancer Center University of Oklahoma Health Sciences Center Oklahoma City, OK United States; 2 School of Public Health University of North Texas Health Sciences Center Fort Worth, TX United States; 3 School of Global Public Health New York University New York City, NY United States

**Keywords:** alcohol use disorder, mobile health, smartphone, ecological momentary assessment, homeless persons

## Abstract

**Background:**

Adults who are homeless are more likely to have alcohol use disorders (AUDs) compared with domiciled adults. Although AUD treatments are commonly available, many factors (eg, transportation limitations and inability to schedule appointments) contribute to low treatment completion rates and low success rates of these interventions among adults experiencing homelessness. Most adults who are homeless own mobile phones; however, no interventions have been developed that use mobile devices to deliver and support AUD interventions for this population. Mobile phone–based AUD interventions may reduce barriers that have limited the use and utility of traditional interventions.

**Objective:**

The aim of this study is to (1) identify variables (eg, affect, stress, geolocation, and cravings) that predict drinking among homeless adults (phase I), (2) develop a mobile intervention that utilizes an algorithm to identify moments of risk for drinking and deliver treatment messages that are tailored to the individual’s current needs in real time (phase II), and (3) pilot test the intervention app (phase III).

**Methods:**

In phase I, adults experiencing homelessness with an AUD (N=80) will complete baseline, equipment, 2-week, and 4-week follow-up visits in person. Participants will be prompted to complete five daily ecological momentary assessments on a study-provided smartphone for 28 days. The smartphone app will collect GPS coordinates every 5 min for the entire 28-day study period. Participants will wear a transdermal alcohol sensor that will objectively measure alcohol use. In phase II, we will use phase I data to develop an algorithm that identifies moments of heightened risk for drinking and develop treatment messages that address risk factors for drinking. Phase III will pilot test the intervention in 40 adults experiencing homelessness with AUD.

**Results:**

This project was funded in June 2018. IRB approval was obtained in October 2018, and data collection for phase I began in February 2019. Phase III data collection is expected to conclude in 2020. To date, 80 participants have consented to the study, and data analysis for phase I will begin in early 2020.

**Conclusions:**

This research will highlight intervention targets and develop a novel intervention for understudied and underserved adults experiencing homelessness with AUD.

**International Registered Report Identifier (IRRID):**

DERR1-10.2196/15610

## Introduction

### Background

An estimated 6.2% of US adults will be homeless at some point in their lifetime [1]. Adults experiencing homelessness have higher rates of disease, greater risk of interpersonal violence, shorter life expectancies, and disproportionately higher health care utilization and costs compared with housed individuals [2-5]. Adults experiencing homelessness are 8 times more likely than adults in the general population to be alcohol dependent [6,7], and the high prevalence (29%-63%) [8-11] of alcohol use disorders (AUDs) is a leading contributor to the higher morbidity and mortality among adults experiencing homelessness. For example, one study found that alcohol was responsible for an estimated 17% of all deaths among homeless adults in Boston, a rate 6 to 10 times greater than in the general population [12].

Although shelter-based AUD treatments are common [13], adherence is typically poor [6]. Homeless individuals experience a number of barriers to receiving AUD treatment, including lack of stable housing [14], fractured social networks [15], and high rates of co-occurring problems [16]. There is evidence that shelter-based interventions are feasible [17] and can decrease drinking [18]; however, keeping clients engaged in treatment is a challenge [19,20]. In an analysis of 15 substance use disorder (SUD) treatment programs for homeless individuals funded by a National Institute on Alcohol Abuse and Alcoholism cooperative agreement [21], dropout rates ranged from 67% to 97.5%. Not a single program was completed by more than one-third of clients. Major reasons for dropout included poor client motivation, a desire to reconnect with family or friends outside of treatment, dissatisfaction with the program structure or environment, and other logistical difficulties.

Relatively little is known about the environmental, cognitive, affective, and behavioral antecedents of alcohol use in adults experiencing homelessness. Alcohol use has most often been examined using traditional lab-/clinic-based assessment methods that are not well suited to capturing the complicated street-level interactions experienced by most adults experiencing homelessness [22,23]. Ecological momentary assessment (EMA), in which handheld devices (eg, smartphones) are used to capture moment-to-moment experience via brief surveys, reduces recall bias and more accurately measures antecedents and correlates of alcohol use in natural settings [22,24-26]. In addition, recent technological advances in smartphone sensors have made it possible to passively collect continuous geolocation data (ie, GPS coordinates) alongside EMA [27]. Thus, momentary changes in key variables can be tracked, studied, and potentially used to initiate real-time interventions and engage clients in treatment.

Multiple studies have identified momentary predictors of smoking relapse [28-33] and have shown that the composite scores based on daily diaries of self-efficacy and motivation are more consistent predictors of drinking outcomes than global measures of self-efficacy and motivation among problem drinkers [34]. Thus, EMAs may be better suited than traditional clinic-based, trait-like measures to identify mechanisms that drive alcohol use.

Although EMA has been used in a variety of populations and for multiple health outcomes, only a few studies [33,35] have collected EMA data in adults experiencing homelessness. Furthermore, few studies have used both EMA and geolocation to assess risk for alcohol use despite the fact that research studies have indicated that most adults experiencing homelessness possess phones with active service (ie, we have conducted two large survey studies of adults experiencing homelessness (Dallas and Oklahoma City) and found that 58.4% of 394 surveyed adults in Dallas (2013) and 71.9% of 589 surveyed adults in Oklahoma City (2016) possessed cellular phones with active service). We are not aware of any studies that have combined this information to estimate risk and intervene in real time to reduce drinking among homeless adults. We believe combining EMA and geolocation data will help improve our understanding of the mechanisms that lead people to drink and pave the way toward more effective and cost-effective alcohol treatments for this high-risk group. This paper describes the rationale and design for a three-phase treatment development study to develop a just-in-time adaptive smartphone intervention (JITAI) to reduce alcohol use in adults experiencing homelessness.

### Objectives

During phase I, we will use smartphones and passive sensing to continuously monitor geolocation and to measure psychosocial variables (eg, negative affect, stress, and urge to drink) and alcohol use in a sample of 80 adults experiencing homelessness enrolled in shelter-based treatment programs. EMAs will be used to examine the moment-to-moment relationship between social cognitive theory (SCT) constructs (eg, affect, abstinence motivation and self-efficacy, alcohol use expectancies, and cravings) [36,37], social-ecological model constructs (eg, current proximity to previous drinking areas or alcohol outlets, social setting, and social support) [38,39], and drinking. We will also assess these constructs as trait-like variables at baseline to examine how trait and state processes interact to influence drinking behaviors. Finally, phase I participants will complete quantitative and qualitative measures at the conclusion of the study. These measures will query about things they liked and disliked about the survey app design and potential intervention components that should be included in the phase III app.

In phase II, we will use this information to develop optimized risk algorithms and develop tailored treatment messages that can be provided before anticipated alcohol use given personal, situational, and environmental triggers (eg, presence of drinking others, location, elevated positive or negative moods, and high stress).

In phase III, we will pilot test the smartphone app for utility, satisfaction, and preliminary effectiveness in another sample of 40 homeless adults enrolled in shelter-based treatments. Algorithm-driven treatment messages will be automatically delivered at the end of EMAs. Phase III participants will complete a qualitative interview that will examine their opinions of the app design and intervention content and ways to improve the app user interface. In phases I and III, self-reported alcohol use will be validated via a transdermal alcohol sensor (ie, Secure Continuous Remote Alcohol Monitor [SCRAM], Alcohol Monitoring Systems, Inc) worn by participants.

Motivational- (eg, derived from motivational interviewing) and self-efficacy- (eg, derived from SCT) themed messages are commonly used in technology-based alcohol interventions [40]. Interventions for AUDs have often drawn from these underlying theories, but mobile interventions have the additional strength of fostering self-regulation through triggering goal salience and re-evaluation of short- versus long-term goals [41]. Recent work has indicated that smartphone apps (eg, the ACHESS app) that incorporate preloaded videos, interactive features, and weekly check-ins can reduce heavy drinking days in alcohol dependent adults [42] and college students [43]. Others have begun to use geolocation data to alert individuals with SUDs about potentially high-risk environments [44-46]. For example, some SMS text messaging interventions for AUD have focused on encouraging self-regulation and planning *before* drinking episodes [47,48]. For those who are enrolled in treatment, the messages can reinforce treatment concepts. For those who are not enrolled in treatment, messages may serve as a primary intervention (or at least a reminder of past concepts) to short-circuit alcohol use before it occurs.

Our central hypothesis is that alcohol use is strongly affected by moment-to-moment risk and protective factors, and we can use EMAs to identify and automatically intervene during moments when people are at high risk for drinking. Our hypothesis is based on preliminary findings from our own studies among homeless [33,49], justice involved [50], and socioeconomically disadvantaged safety net hospital patients [31,32]. If effective, this smartphone treatment app could significantly improve treatment engagement, drinking outcomes, and quality of life among adults experiencing homelessness with AUDs.

## Methods

### Setting

All phases of the project will be conducted at a large homeless shelter located in Dallas, Texas. The shelter provides multiple services, including meals, mental health and substance abuse counseling, care management, housing placement, and job readiness training to approximately 85% of all homeless adults in Dallas County each year. The shelter conducts approximately 366 new intakes each month, of which approximately 32% self-report current *problems with alcohol*. See Table 1 for characteristics of the shelter population [51].

**Table 1:** Characteristics of people enrolled in the Bridge Homeless Recovery Program (N=394).

Characteristics		Value
Sex (female), n (%)		111 (28.2)
**Race/ethnicity, n (%)**		
	Black	247 (62.7)
	White	102 (25.9)
	Latino/Hispanic	24 (6.1)
	Multiracial/other	21 (5.3)
Age (years), mean (SD)		43.9 (11.8)
**Socioeconomic characteristics**		
	Education (years), mean (SD)	11.9 (1.8)
	Insured, n (%)	93 (23.6)
	Employed, n (%)	39 (9.9)

### Eligibility Criteria

We will include relatively few exclusion criteria so that the sample will be as representative of the population as possible. Homeless individuals at the shelter will be included in the study (N=80 for phase I and N=40 for phase III) if they (1) receive a score of 8 or above on the Alcohol Use Disorders Identification Test [52] (a cutoff score suggesting hazardous and harmful alcohol use), (2) report consuming at least one standard drink of alcohol in the past week, (3) are receiving services at the shelter, (4) are willing and able to complete the baseline and follow-up visits, (5) score ≥4 on the Rapid Estimate of Adult Literacy in Medicine-Short Form indicating >6th grade English literacy level (ie, a 7th grade reading level is necessary to complete assessments), and (6) score ≥24 on the Mini-Mental State Exam indicating no substantial cognitive impairment. People with circulation problems, neuropathy, deep vein thrombosis, leg ulcers, tendonitis, diabetes, pregnancy, history of swelling, or nickel or other metal allergies will be asked to consult a shelter-based medical professional before wearing the SCRAM bracelet. Individuals will be excluded from participating if they indicate that they would be uncomfortable wearing the SCRAM bracelet for 4 weeks. Individuals will be excluded from participating in phase III if they participated in phase I.

### Participant Recruitment and Procedure

Homeless adults who are receiving services at the shelter will be given a flyer that briefly outlines this study. Interested individuals will be scheduled for a screening visit to determine study eligibility. Before screening, participants will be informed that shelter services are not contingent upon study enrollment. Those who remain interested will complete the informed consent process before screening. Once eligible, participants will complete a baseline assessment, equipment visit, 2-week follow-up, and 4-week follow-up visits in a private office at the shelter. At the baseline visit, participants will complete baseline questionnaires. Approximately 3 to 7 days after the baseline assessment, participants will return for the equipment visit where they will receive the study smartphone (including instructions on how to complete the phone assessments) and be fitted with the SCRAM bracelet. All study phones loaned to participants will have a data plan which includes unlimited calls and texts and 2 GB of data per month. The app will prompt surveys, collect data, and provide intervention content even when offline (ie, no internet connection is needed for the app to work). Whenever cellular service or Wi-Fi are available, study data are automatically uploaded to the study server.

**Figure 1:**
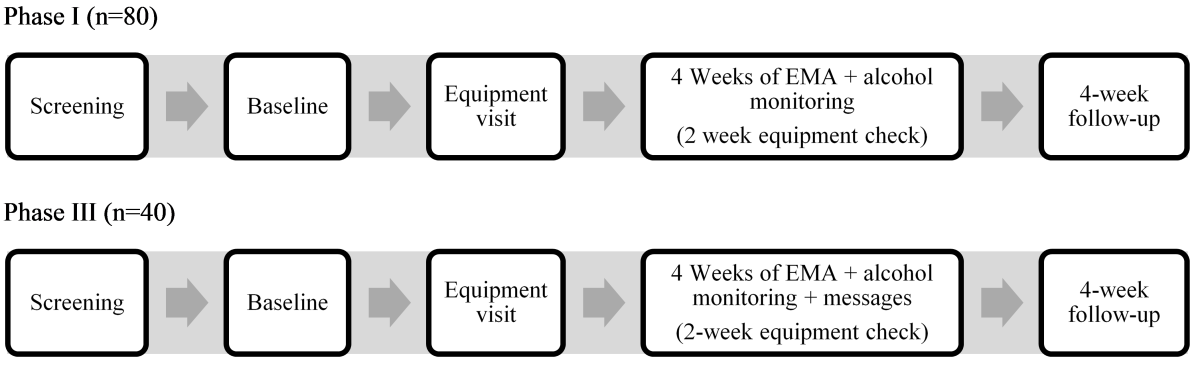
Phase I and III flowcharts. EMA: ecological momentary assessment.

Figure 1 shows the study flow for phases I and III.

### Measures

#### Baseline Measures (In-Person)

In-person assessments will be administered at the baseline and 4-week visits. These measures will be used to describe the sample, identify variables that predict drinking, and help develop the treatment messages. At the baseline visit, a comprehensive locator form will be used to identify and collect multiple ways to contact participants (eg, personal email address, Facebook page, and family members’ phone numbers and addresses) to reduce loss to follow-up. Data will be collected on tablet computers using Questionnaire Development System (QDS) software by NOVA Research Company (Silver Spring, Maryland). QDS utilizes a computer-administered self-interview format (ie, audio computer-assisted self-interviewing), which reduces data entry errors and the need to retain paper copies of questionnaire data. Each item appears on the computer screen while the program reads the item. Participants touch the screen to select their answers after QDS reads each item. In past studies, participants have reported few problems using the QDS program, including those with no computer experience. Trained research staff will be available to help participants who have difficulty. The baseline visit takes approximately 1 hour to complete, and the 4-week visit takes approximately 50 min to complete (see Table 2 for measures).

**Table 2:** In-person assessment measures.

Category	Measure
Background/history	Locator Form Demographic Information Questionnaire^a^ Subjective Social Status [53] Brief Homelessness Questionnaire Homelessness Timeline Follow-Back [54]^a^
Health/mental health	Short Form Health Survey (SF-12) [55] Health Related Quality of Life [56] Self-Rated Health [57] Tobacco Questionnaire Inadequate Sleep [58] Time Line Follow-Back (past month alcohol) [59] Short Inventory of Consequences [60]
Stress/affect	Personal Victimization [61] Perceived Stress Scale-Short Version [62] Urban Life Stress Scale [63] Depression [64]
Interpersonal/intrapersonal	Interpersonal Support Evaluation List [65] Brief Coping Orientation to Problems Experienced (COPE) [66] Religious Participation
Treatment satisfaction	Just-in-Time Adaptive Intervention Satisfaction Survey (quantitative and qualitative components)^b^ System Usability Scale^b^ [67]

^a^Baseline only.

^b^Follow-up only.

### Ecological Momentary Assessment (Phone-Based Measures)

EMA items completed on the phone (see Table 3) will assess SCT constructs (eg, affect, abstinence motivation and self-efficacy, expectancies, and cravings) and social-ecological model constructs (eg, proximity to previous drinking areas, social setting, and social support) to identify key variables and time- and location-dependent fluctuations in variables, which will be used to predict study outcomes. Most of these items have been used in our previous studies and studies from other labs [22,68]. Three types of EMAs will be used: daily diary, random sampling, and event sampling. Daily diary and random sampling EMAs will be initiated by the phone. The phone will audibly and visually cue these EMAs for 30 seconds. If the participant has not responded after 5 prompts, the assessment will be recorded as missed. Event sampling is initiated by participants if/when they consume their first drink in a day. On average, random and event sampling assessments take 2 min to complete, and daily diary assessments take less than 5 min to complete.

#### Daily Diary

**Figure 2:**
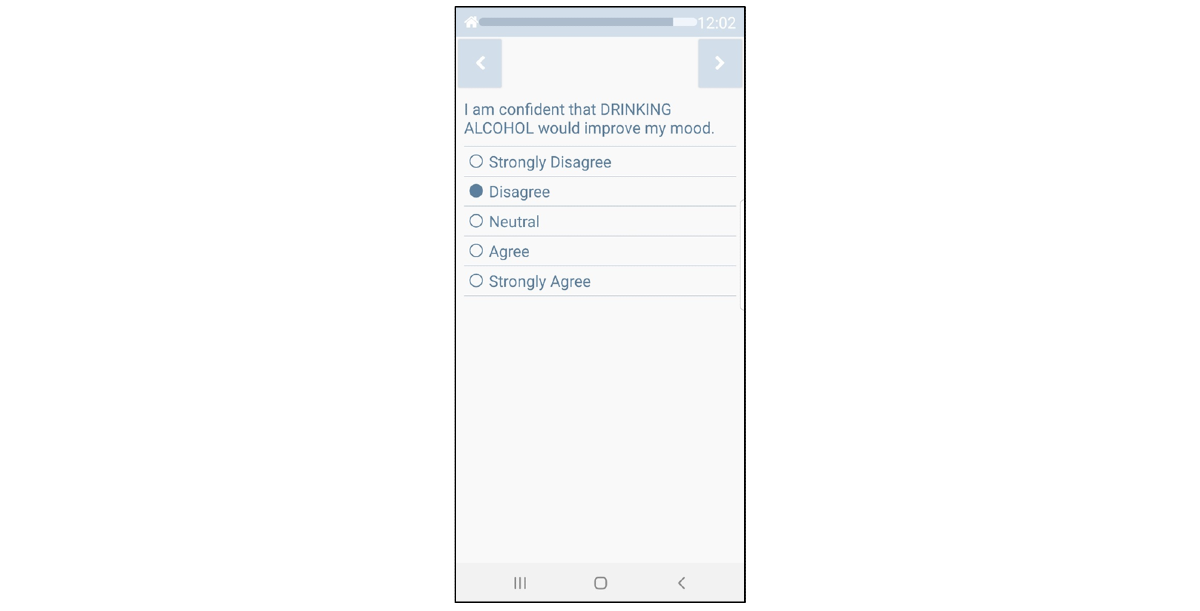
Smart-T alcohol example question.

Daily Diary EMAs will be completed each day 30 min after the participant’s self-reported wake time; questions will ask about the previous day (ie, “yesterday”) and current (ie, “right now”) experiences. Alcohol consumption will be assessed with the item “Did you drink any alcohol yesterday?” If the participant answers “yes,” he/she will be prompted to indicate the number of standard drinks that were consumed. EMA reports have generally been seen as valid measures of drinking, even when participants are intoxicated [69,70]. See Figure 2. Additional items will assess sleeping arrangements from the prior night (example answer options: friend or family member’s house or apartment, homeless shelter, jail, car, outside on the street), quality of sleep the previous night, social support and types of social interactions, stressors, other substance use, and substance abuse treatment attendance (see Table 3).

**Table 3:** Ecological momentary assessment (EMA) measures.

Type of EMA	Measure
Daily diary	Sleeping arrangements Social support and interactions Treatment attendance Current stressors and perceived stress Alcohol consumption Other substance use
Core/random/event sampling	Affect/stress Urge to drink Alcohol availability Social setting/location Recent alcohol consumption Expectancies Abstinence motivation Abstinence self-efficacy New/ongoing stressful events^a^ Reasons for drinking^a^ Modified conflict tactics scale

^a^Drinking assessments only.

#### Random Sampling

Participants will be prompted at random times to complete EMAs 4 times each day, scheduled to occur during the participant’s normal waking hours. Participants will rate their affect by indicating the extent to which they agree or disagree with each of 13 statements at the moment: *I feel irritable, happy, content, angry, sad, worried, miserable,* *restless, stressed, hostile, calm, bored, and* *depressed* (most items are from the circumplex model of affect [71]). In addition, participants will describe their current environment (eg, shelter, work, outside, or bar) and social setting (eg, alone, with others, or with others who are drinking). Alcohol urges (ie, “I have an urge to drink alcohol”; answer options range from strongly disagree to strongly agree), alcohol availability (ie, “Alcohol is available to me”; answer options range from not at all to easily available), drinking start/stop time, recent drinking, expectancies, motivation for abstinence, and abstinence self-efficacy will also be assessed during random sampling.

#### Event Sampling

Participants will be instructed to click the “I am About to Drink” or “I Just Drank” buttons if/when they have their first drink of the day. Drinking assessments will include all items from the random assessments and will query the reinforcing value of the drink or drinks and causes of the drinking episode.

#### Geocoding

The smartphones will be programmed to collect geolocation (ie, latitude, longitude) coordinates every 5 min.

### Transdermal Alcohol Monitor

Biosensors can provide a continuous estimate of blood alcohol concentration (BAC) based on the concentration of alcohol in skin perspiration (Swift et al, unpublished data, August 1993) [72-78]. The device with the most extensive evaluation is the SCRAM bracelet (Alcohol Monitoring Systems Inc, Littleton, Colorado), which is worn on the ankle. SCRAM has an electrochemical sensor that samples the vapor from the skin every 30 min and stores readings for later retrieval. Peak transdermal alcohol concentration (TAC) is highly correlated with peak BAC, and self-reported volume of alcohol consumed is correlated with TAC estimates [79,80]. A recent project was able to detect 93.0% of self-reported heavy drinking episodes (≥5 drinks) using SCRAM data [81]. Data from the SCRAM are uploaded to SCRAMnet via USB cable and downloaded to a personal computer for analysis. The SCRAM is water resistant and cannot be removed without cutting the strap. To help address any stigma of wearing the device, participants will be provided with a laminated card that confirms their participation in the study and will be given a large (ie, bariatric) sock to wear over the equipment to make it less visible and more comfortable. At follow-up, participants will be asked about their experiences wearing the SCRAM.

### Smartphone Hardware

A Samsung Galaxy J3 smartphone (or equivalent) will be loaned to each participant so that they may complete EMAs during the study. Participants enter data by touching their response on the screen (see Figure 2 for an example EMA item). Participants will be able to call (eg, if they have problems completing EMAs) and receive calls from research staff through the smartphone free of charge. The phone app encrypts data as they are collected and uploads data to the server multiple times per day.

### Smartphone Programming

The mobile health (mHealth) Shared Resource at the NCI Designated Stephenson Cancer Center will provide the programming services for this project. The mHealth resource employs a program manager, 2 research technicians, and 4 senior programmers who develop and maintain Web and mobile apps and relational databases. Apps are developed using the Insight platform, which consists of two components: a content management system (CMS) where researchers log in to CMS to create EMA/JITAI content and set EMA schedules, and a smartphone app shell. Once content is created, researchers transfer study materials into the smartphone app shell, greatly reducing the amount of time needed to create and deploy their smartphone app. The corresponding author is the Scientific Director of the mHealth Shared Resource.

### Smartphone Training

**Figure 3:**
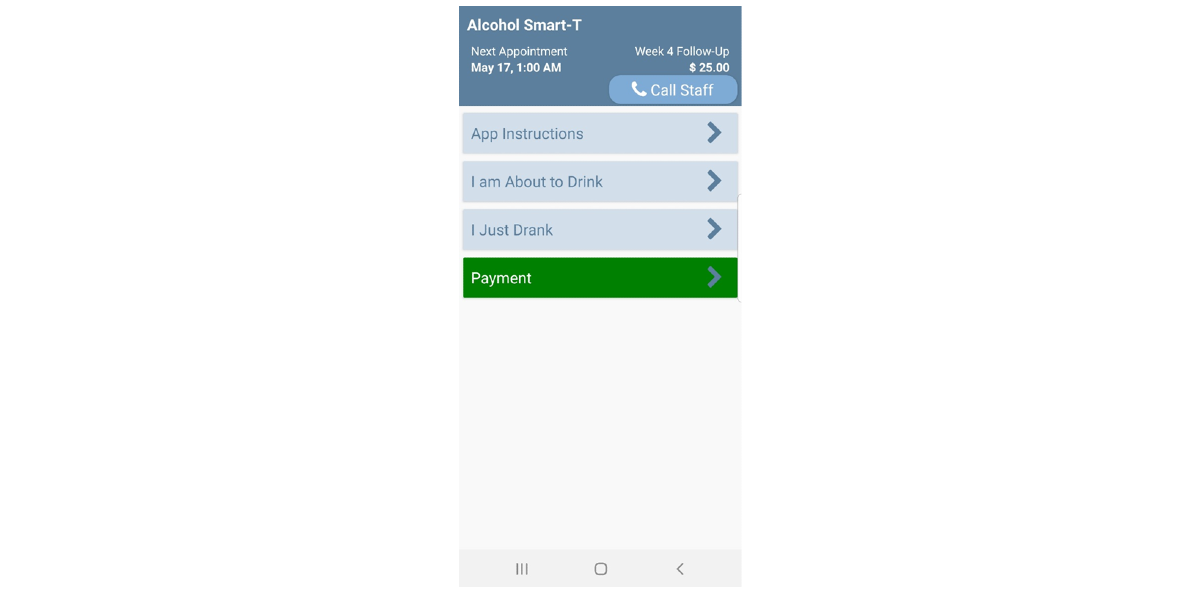
Smart-T alcohol phase I home screen.

Participants will watch a brief step-by-step video tutorial at the baseline visit that demonstrates how to use the app. This video will be loaded onto the app home screen (see “App Instructions” in Figure 3) so participants may view it at any time. The video will discuss how to complete EMAs and how to use the “Call Staff” and “Payment” button/options. We have achieved high EMA adherence rates (ie, 82%-87% of all EMAs completed) using similar protocols in previous samples of socioeconomically disadvantaged people (eg, homeless smokers and safety net hospital patients) [31,49].

### Compensation

Participants will be compensated with a US $25 gift card for completing the baseline assessment, US $25 for completing the 4-week assessment, up to US $25 in gift cards per week for completing EMAs (prorated based on percent completed), and US $25 for returning the phone and SCRAM in good condition at the end of the study. Specifically, those who complete 50% to 74% of EMA assessments each week will earn US $15 in gift cards, those who complete 75% to 89% of assessments will earn US $20 in gift cards, and those who complete 90% or more of their EMAs will earn US $25 in gift cards (payable at the 2- and 4-week visits). Participants who complete less than 50% of the EMA prompts will not receive any compensation for the EMA component for that period. The phone shows the percent of EMAs completed. Overall, a participant can receive US $25 at the baseline visit, up to US $50 at the 2-week equipment check visit, and up to US $100 at the final visit.

### Phone Data Loss Prevention

To overcome potential loss of data if participants lose the study phone, phones will be programmed to connect to our secure server each day to upload encrypted data. This will minimize EMA data loss and allow researchers to monitor each participant’s EMA completion rate and intervene when the rate is low. Importantly, EMA data are password-protected and encrypted on the study phone. Thus, study data are only accessible by the research team. If a phone is lost, it will be remotely wiped. We will provide one replacement phone if the participant has completed at least 50% of assessments for 1 week.

### Phase III App

**Figure 4:**
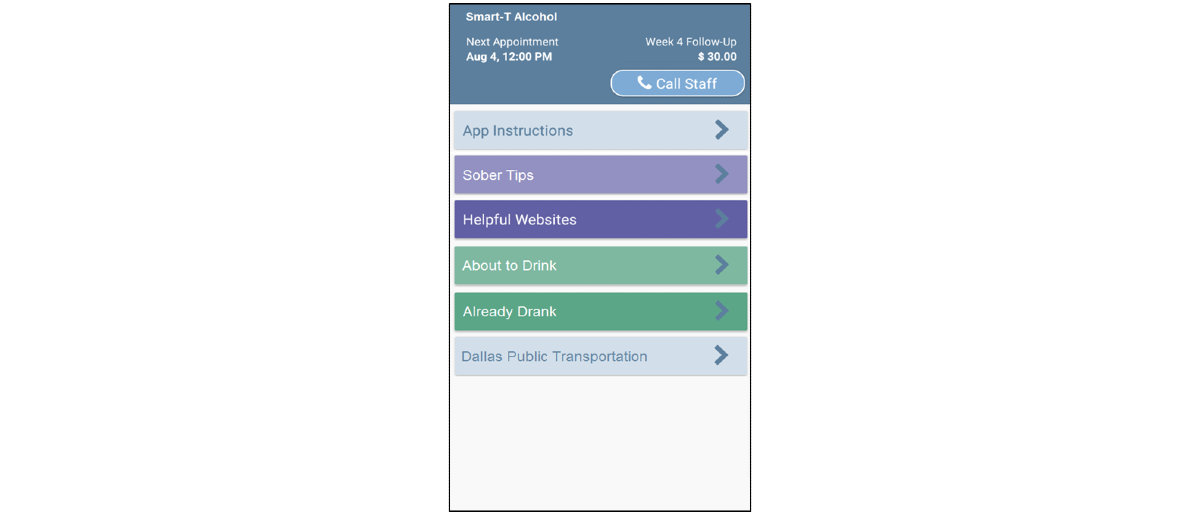
Anticipated Smart-T alcohol phase III home screen.

The phase III intervention app will have multiple components including (1) an on-demand “Tips” function/button, (2) a “Helpful Websites” function/button, (3) a “Call Staff” function/button, and (4) an algorithm that will use recent EMA responses and geolocation to assess current risk for alcohol use and automatically push relevant tailored messages to participants. The phone will record date/time when each of the components is accessed. See Figure 4 for the anticipated phase III home screen.

#### Sober Tips Function

Clicking this on-demand option will open a new window that will enable individuals to get useful tips related to “Benefits of Sobriety,” “Motivational Messages,” “Alcohol Refusal Skills,” and “Managing Urges.” The tips will be developed using strategies from previous motivational and skills-based interventions, such as the MAPIT [50], m.chat [82], and Smart-T [31] studies. For instance, when the “Managing Urges” tips option is clicked, participants will receive a suggestion on how to cope with their current urge to drink. This function will enable participants to access tailored messages at any time. Participants may view additional tips by clicking the “Next” button. Type and number of tips viewed will be recorded by the smartphone. Other topics for tips will be identified via examination of phase I participants’ survey and EMA data.

#### Helpful Websites Function

Clicking this option will open a menu of useful websites (eg, Dallas public transit routes, Google maps, and online support groups such as Alcoholics Anonymous).

#### Call Staff Function

Clicking this option will connect participants to study staff if they encounter problems with the study phone and for scheduling/rescheduling follow-up appointments.

#### Risk Algorithm

The algorithm used to guide the just-in-time treatment messages will be similar to the algorithm that was developed for the Smart-T smoking cessation app [31]. Specifically, the algorithm will estimate risk for alcohol use using variables identified in phase I. In Smart-T, we attempted to develop risk algorithms that could predict smoking at 8, 12, and 24 hours before the lapse, but these algorithms were far less sensitive than the 4-hour lapse prediction algorithm. The resulting Smart-T algorithm combined six EMA variables (ie, urge, stress, cigarette availability, alcohol use, motivation to quit, and proximity to others smoking) to successfully predict 80% of all smoking lapses within 4 hours of lapse occurrence (false positive rate=17%) [31,32].

In phase III, smartphones will push tailored messages based on the momentary risk algorithm score at the end of each EMA. We anticipate that participant responses that indicate low risk for imminent alcohol use (eg, within the next 4 hours) will prompt delivery of level 1 messages. Level 1 messages will primarily focus on increasing motivation for abstinence, avoiding people/places/things that may trigger alcohol use, benefits of sobriety, advice on ways to escape high-risk situations, and advice to seek support from others [50]. These messages will complement the treatment themes for those who are in an alcohol treatment program. Level 2 messages will be delivered at the end of EMAs if the algorithm determines that there is heightened risk for imminent (eg, within the next 4-8 hours) alcohol use. These messages will focus on in-the-moment distraction, reframing, immediate help-seeking, planning, and other tools to reduce craving. The highest rated indicator/trigger of alcohol use in that moment will be the topic of the level 2 tailored treatment messages. For example, if a participant reports low motivation for sobriety and average ratings on the other variables, they will receive a message that aims to boost motivation. An example may read: “You said that family was an important reason for staying sober. You’re looking forward to a better life!” Likewise, if exposure to drinkers is an identified alcohol use trigger and a participant reports that he/she is near individuals who are consuming alcohol, he/she may receive a tailored suggestion on how to escape that high-risk situation, such as “You said that removing yourself from a situation was often helpful in managing cravings. Some people decide to get out of the situation, before they are tempted to drink.” Participants will receive level 3 messages when they report recent drinking. Level 3 messages will focus on reframing the drinking episode as a learning experience and considering strategies for handling the situation differently in the future. We will draw from best-practice recommendations around message content and tone [83]. Our past interventions have contained hundreds of possible message combinations, depending on a person’s baseline profile and current responses.

At the completion of phase II, we will have a working app that includes all components described above. The app will utilize EMA data (eg, location, time of day, urge to drink, affect [positive and negative], motivation, abstinence self-efficacy, and nearby presence of others who are drinking) to calculate risk and automatically intervene to reduce alcohol use in real time.

### Statistical Analyses

Our assessment protocol is designed to capture diurnal patterns of experience and behavior within, between, and across days. Thus, data will have multiple time scales nested within individuals, ranging from every 5 min to monthly (ie, 288 geolocation assessments per day, 48 TAC readings per day, 5 prompted EMAs per day, participant-initiated drinking event EMAs, and monthly in-person assessments). Time and geolocation will be used as the variables to record risk and protective variables in calibrating one’s risk. Traditional generalized linear mixed models; machine learning algorithms, such as elastic net penalized cox proportional hazards regression [84], or random forests [85], as well as spectral and dynamic modeling analyses, if feasible, will be used to identify predictors of study outcomes, model auto-regressive cyclical patterns, and capture intra- and interpersonal risk processes. We will divide the sample into training and testing datasets to validate the algorithm. Examples of planned analyses include (1) testing if alcohol urges or measures of affect predict daily drinking status, (2) testing if protective factors (eg, social support, positive interpersonal interactions, and time and location) predict alcohol use, and (3) testing if parameters of key variables (ie, intercept; slope, eg, increasing urges to drink over time; quadratic term; and volatility, eg, the symptom scatter or the ups and downs of urges over time) predict alcohol use.

Different levels of risk across individuals will also be included to examine how intraday risk gets intensified or ameliorated by personal trait-level variables (eg, sex, psychosocial resources, stress/adversity, and negative mood). We expect that analyses of SCT and ecological constructs (eg, affect, expectancies, self-efficacy, and proximity to drinking areas), gathered during random EMAs and breadcrumb trail geolocation, will identify patterns that predict drinking in near real time. In addition, EMA data will allow us to examine other important methodological questions such as (1) agreement between SCRAM, Timeline Follow-Back, and EMA reports of alcohol use and (2) the effect of episodic events (eg, exposure to violence or other stressors) on self-efficacy and mood and what impact that has on alcohol use. We will use SCRAM-detected alcohol use to explore the utility of SCRAM for validation of self-reported use events. We will use a macro developed by Barnett and colleagues [86] to interpret TAC data. We anticipate some missing data because of SCRAM bracelet malfunction, participant nonadherence, or participant attrition. In Barnett’s work, data loss because of bracelet malfunction occurred on less than 5% of days of data collection, though we expect data loss to be somewhat higher in this study because of the nature of the population.

Questions similar to those listed in Table 4 will be used to assess the feasibility, acceptability, and usability of the phase III app. We will compare phase III participants (ie, EMAs and app features including tailored treatment messages) to phase I participants (ie, EMAs only) to examine the preliminary effectiveness of the app. Specifically, we will compare phase I and phase III participants’ percent drinking days (PDD) and percent heavy drinking days (PHDD; ≥5 drinks for men, ≥4 drinks for women) using generalized linear models with an appropriate link function to accommodate outcome distributions. We will consider the study phase as the parameter of interest estimating the treatment effect, adjusting for relevant covariates (eg, gender, race, and baseline AUD severity). Exploratory analyses will examine the effect of specific types of treatment messages on intervention targets. For example, we will examine whether phase III participants’ urges to drink are attenuated in EMAs that follow urge messages, and if postmessage reductions in urge are different/greater than that of phase I participants.

**Table 4:** Example acceptability items.

Question	Answer range
Overall, how helpful were the messages at the end of each assessment?	0=Not at all, 5=Extremely
Did the assessments and messages help you to make decisions that supported sobriety?	0=Definitely no, 5=Definitely yes
Overall, how helpful has the smartphone app been in helping you stay sober?	0=Not at all, 5=Extremely
How likely would you be to recommend the app to a friend?	0=Not at all, 5=Extremely

## Results

The North Texas Regional Institutional Review Board approved the protocol as presented in this study in October 2018. The phase I smartphone app has been developed (see Figure 3), and data collection began in February 2019. Phase III data collection is expected to conclude in 2020. To date, 80 participants have consented to the study, and data analysis for phase I will begin in early 2020.

## Discussion

### Phase I

Phase I will advance previous research by identifying trends in behaviors, cognitions, and geolocation that predict subsequent drinking. This information will guide the development of JITAI in phase II that will be pilot-tested in phase III. We expect in-person assessments to show how psychosocial resources, stress/adversity, negative affect, and exposure to other drinkers and drinking locations affect alcohol use. We expect that analyses of SCT and ecological constructs (eg, affect, expectancies, self-efficacy, and proximity to drinking areas), gathered during EMAs and breadcrumb trail geolocation, will identify patterns that predict drinking in near real time. Findings from these analyses will identify additional targets for the intervention that will be developed in phase II.

Phase I will provide the foundation for one of the first smartphone interventions to be evaluated among adults who are homeless. As this population often lacks access to traditional intervention programs, the use of smartphone technology has tremendous potential to remove and attenuate barriers to service utilization for this at-risk population. Regardless of our initial intervention results, this work will provide valuable data on the array of risk and protective factors at multiple levels across multiple contexts affecting decision making and alcohol-use behavior in homeless adults.

### Phases II and III

During phase II, we will create a “real time” drinking risk algorithm that can be used to deliver tailored treatment messages based upon current estimated risk of alcohol use. The integration of individual-level environmental exposure data alongside state-of-the-art EMA methodology sets this project apart from past studies. A strength of this project is that it does not rely solely on self-reported data but also embraces the “ecological” part of EMA by linking an individual’s behavior to the real-time environment in both time and location. Continuous “bread-crumb trail” geo-tracking provides a within-person control by documenting a person’s behavior with and without the presence of risk-promoting factors [87-89].

Treatment tailoring is most often done using participant characteristic or characteristics that are assessed at the baseline visit (eg, sex, and level of dependence). The proposed intervention will take this approach one step further by tailoring messages based on real-time risk for alcohol use. Phase III will test the initial efficacy of a smartphone app that assesses risk for alcohol use and automatically intervenes with tailored, theory-based treatment messages based on level of risk. The additive design of this project provides an analysis framework that will allow us to preliminarily examine the comparative effectiveness of the automated treatment messages triggered by our (phase II algorithm) relative to the calibration sample recruited in phase I. We expect that phase III participants will rate the app as helpful and useful and to report that it helped them to make decisions that were supportive of abstinence. We also anticipate preliminary evidence that phase III participants will demonstrate lower PDD and PHDD compared with phase I participants.

### Limitations

Several study limitations warrant mention here. First, the intervention app that will be developed and tested during phase III may not be applicable to those with low literacy and cognitive impairment (eg, a 7th grade reading level is required to read and understand the smartphone-based assessments and intervention content). Second, only those who have access to a smartphone and access to electrical outlets to charge their phone will benefit from this type of intervention. It is important to note that research from our laboratory in two cities (ie, Dallas and Oklahoma City) has indicated that most adults experiencing homelessness possess phones with active cellular service. Finally, it is possible that EMA or SCRAM monitoring may have independent effects on drinking, especially given the frequency of assessment in this study. Some studies have found that self-monitoring can lead to changes in drinking, even without an “intended” intervention [90,91]. However, other studies have found only small, time-limited effects of frequent assessment, and in a study of college drinkers, frequent self-report and SCRAM monitoring did not strongly affect drinking behavior [92]. Nevertheless, we acknowledge that the design of this study will test the difference between assessment alone versus assessment + intervention messages and that potential therapeutic effect of monitoring needs to be studied in future studies.

### Future Directions

This study will identify real-time antecedents of drinking among adults who are homeless, develop algorithms to predict risk of alcohol use and tailored treatment messages, and provide pilot data on the efficacy of JITAI. Many people do not respond adequately to currently available treatment formats. This is particularly evident in underserved populations such as homeless adults, where treatment adherence tends to be poor. This project will gather valuable data on the risk and protective factors that affect drinking among adults experiencing homelessness. This information will be critical to developing other innovative treatments for this understudied and underserved population.

## Abbreviations

AUD: alcohol use disorder
BAC: blood alcohol concentration
CMS: content management system
EMA: ecological momentary assessment
JITAI: just-in-time adaptive intervention
mHealth: mobile health
PDD: percent drinking days
PHDD: percent heavy drinking days
QDS: Questionnaire Development System
SCRAM: Secure Continuous Remote Alcohol Monitor
SCT: social cognitive theory
SUD: substance use disorder
TAC: transdermal alcohol concentration
